# Haemolytic-Uraemic Syndrome as a Sequela of Diarrhoeal Disease

**DOI:** 10.3329/jhpn.v30i3.12288

**Published:** 2012-09

**Authors:** Christa L. Fischer Walker, Jennifer A. Applegate, Robert E. Black

**Affiliations:** Department of International Health, Johns Hopkins Bloomberg School of Public Health, Baltimore, MD 21205, USA

**Keywords:** Colitis, Haemorrhagic, Diarrhoea, *Escherichia coli*, Enterohaemorrhagic, Haemolytic-Uraemic Syndrome, Review literature, *Shigella dysenteriae*, Systematic review

## Abstract

Haemolytic-uraemic syndrome (HUS) is a serious sequela of diarrhoea and results in a high mortality rate. This systematic review aimed at estimating the proportion of HUS cases that are linked to prior infection due to Shiga toxin-producing *Escherichia* coli (STEC) or *Shigella dysenteriae* type 1. A systematic review of the existing literature was done to identify cohort and case-control studies that examined the relationship between STEC and *S. dysenteriae* type 1 and HUS. After screening 2,516 articles, 11 studies were found that met the inclusion/exclusion criteria. Findings of case-control studies suggest that 60.8% of the HUS cases may be attributable to a previous infection with STEC. In cohort studies, 7.8% of participants with STEC and 8% of participants with *S. dysenteriae* type 1 developed HUS during follow-up. HUS is linked to diarrhoea due to both STEC and *S. dysenteriae* type 1. Thus, preventing infections caused by both pathogens is critical for the prevention and control of HUS, especially in areas where timely and effective treatment is not available.

## INTRODUCTION

Haemolytic-uraemic syndrome (HUS) is a disorder clinically associated with low platelet count, acute renal failure, and non-immune haemolytic anaemia (1,2). HUS is known to be sequelae of two gastrointestinal pathogens: Shiga toxin-producing *Escherichia coli* (STEC), also known as enterohaemorrhagic *E. coli* (EHEC), and *Shigella dysenteriae* serotype 1 (*S. dysenteriae* type 1) (3,4). HUS can be severe with the majority of patients requiring red blood cell transfusions. Other complications include neurological impairment and renal failure needing dialysis. Despite improvements in intensive care facilities and availability of dialysis in developed countries, there remains a 3-5% case-fatality rate for patients in the acute phase of Shiga-toxin (Stx)-induced HUS (5,6). In developing countries with more limited care, the prognosis is likely to be much worse.

It is thought that the incidence of HUS in children and adolescents is higher than in adults, and HUS is recognized as the leading cause of acute renal failure in childhood. Globally, STEC are considered the primary cause of haemorrhagic colitis (7). However, the past estimates have been based on 1-2 study(ies), not a formal review of the literature. We conducted a systematic review of published studies to estimate the proportion of HUS cases that may be attributed to STEC and *S. dysenteriae* type 1 from all regions of the world.

## MATERIALS AND METHODS

We performed searches in PubMed, Scopus, and Embase/Medline for studies published during January 1980–August 2011 that investigated the relationship between STEC or *S. dysenteriae* type 1-associated infection and subsequent HUS. In PubMed, we searched using combinations of the following Medical Subject Headings (MeSH): ‘*Shigella*’, ‘Shiga Toxins’ and ‘Hemolytic-Uremic Syndrome’ and the key words: ‘*Shigella*’, ‘*S. dysenteriae* type 1’, ‘Shiga toxins’, ‘Shiga Toxin’, ‘Shiga’, ‘VTEC’, ‘STEC’, ‘Hemolytic Uremic Syndrome’, ‘Haemolytic Uraemic Syndrome’, ‘Gasser's Syndrome’, ‘Gasser Syndrome’, or ‘HUS’. For Embase/Medline and Scopus, we used a combined search of the following concepts and their Emtree synonyms: Shiga Toxins, *Shigella*, and Hemolytic Uremic Syndrome. STEC, EHEC, VTEC, and SLTEC are equivalent terms that refer to *E. coli* strains that produce one or more toxin(s) of the Shiga-toxin (Stx) family; thus, we included all the terms in the final search.

We included case-control and cohort studies in this systematic review. For cohort studies, the primary outcome was the development of HUS in persons with laboratory-confirmed STEC or *S. dysenteriae* type 1-associated infection. For case-control studies, the primary outcomes were rates of laboratory-confirmed infection due to STEC or *S. dysenteriae* type 1 in HUS cases (typical and/or atypical) and non-HUS controls. In the case of multiple control groups, we used all available controls for the final calculations.

Studies were included if serum and/or stool samples were collected during the acute phase of HUS within 24-48 hours of patient's admission to the hospital and no longer than four weeks after admission. Enzyme-linked immunosorbent assay (ELISA), used in conjunction with immunoblotting, may detect serum antibodies produced by STEC (8,9). The ELISA test may provide evidence of infection when faecal STEC cannot be detected (8,9). Studies were excluded if stool or serology cultures were not obtained to confirm *S. dysenteriae* type 1 or STEC in both cases and controls. We also excluded case-control studies with fewer than 15 HUS cases and cohort studies, including HUS cases that developed more than two weeks after a confirmed infection with STEC or *S. dysenteriae* type 1. We also excluded any studies that focused on HUS presentation in special populations to limit heterogeneity among study subjects. For example, we excluded case-control studies that included malnourished or HIV-positive children.

**Table.** Proportions of STEC infections in HUS case-control studies

				HUS cases		Controls	
Author	Year	Country	Duration of study	Study population with HUS	Positive for STEC (%)	Population	Positive for STEC (%)
Cordovéz* (14)	1992	Chile	1988-1989	20	6 (30.0)	38	2 (5.3)
Gianviti (15)	1994	Italy	1988-1992	68	49 (72.0)	58	2 (3.0)
Greatorex (16)	1994	USA	1989-1992	27	23 (85.2)	47	3 (6.4)
Jure (17)	1998	Argentina	1994-1996	19	12 (63.2)	17	0
Karmali (18)	1985	Canada	1980-1983	40	24 (60.0)	40	0
Kishore (19)	1992	India	NA	28	19 (67.9)	25	0
Kleanthous* (20)	1990	United Kingdom	1985-1988	185	58 (31.3)	148	9 (6.1)
Lopez (21)	1989	Argentina	1986-1988	51	34 (66.7)	64	13 (20.3)
Total				438		437	
Median (IQR)					64.95 (52.8-68.9)		4.15 (0-6.175)

*No serology tests;

HUS=Haemolytic-uraemic

syndrome;

IQR=Interquartile range;

NA=Not applicable;

STEC=Shiga toxin-producing *Escherichia coli*

The definitions of HUS were based on the currently-accepted criteria for diagnosing HUS, which include haemolytic anaemia, acute renal failure, and a low platelet count (1,2). Studies were excluded if they did not explicitly state or cite criteria for the diagnosis of HUS. We reviewed all titles and abstracts to identify eligible studies and included articles written only in English. Full manuscripts were obtained for potentially-eligible studies.

For case-control studies, we calculated the median and interquartile range (IQR) for the HUS-positive cases and controls. We used Microsoft Excel to calculate the medians and IQR for each group (10). For cohort studies, we calculated the incidence of HUS among those exposed to STEC or *S. dysenteriae* type 1 and the non-exposed (where applicable).

## RESULTS

We screened 2,516 potential studies for inclusion in the review. After applying the inclusion and exclusion criteria, 11 studies were included for abstraction. We found one prospective cohort study that included persons with laboratory-confirmed *S. dysenteriae* type 1 (11) and two that included STEC-infected persons (12,13). In these studies, subjects were followed prospectively for the development of HUS (11-13). We also included eight retrospective case-control studies that tested for STEC infection in HUS-confirmed cases and non-HUS controls (14-21). Overall, the retrospective case-control studies yielded 438 HUS cases and 437 controls (Table). Six studies were conducted in developing countries (11,12,14,17,19,21) and five in developed countries (13,15,16,18,20).

In the prospective cohort study by Khin-Maung-U *et al*., four cases of HUS were observed in the group with *S. dysenteriae* type 1 (n=50), yielding an incidence of eight HUS cases per 100 cases of *S. dysenteriae* type 1 (11).

Lopez *et al*. followed 93 children with laboratory-confirmed STEC and found that eight (8.6%) developed HUS during the study period (12). McPherson *et al*. enrolled a cohort of 114 persons positive for STEC and 304 subjects without STEC and followed them prospectively to determine the incidence of HUS. Of the 114 study participants with STEC, eight patients (7%) developed HUS during follow-up. None of the subjects without STEC developed HUS during the study period (13). When combined, these studies have a median HUS incidence of 7.8% among those children infected with STEC (12,13).

For retrospective case-control studies, rates of STEC-associated infection among HUS cases ranged from 30% to 85.2% while infection rates among controls ranged from 0 to 20.3%. The median positive for STEC among HUS cases was 64.95% (IQR 52.825-68.925), and the median positive for STEC among controls was 4.15% (IQR 0-6.175). These findings suggest that 60.8% of the HUS cases may be attributable to a previous infection with STEC.

## DISCUSSION

In this review, we found 11 studies that met our inclusion/exclusion criteria and measured the association of STEC or *S. dysenteriae* type 1 with HUS. We initially searched for cohort studies because it is the only study design that can provide true incidence of HUS following an infection with *S. dysenteriae* type 1 or STEC. However, pathogen-specific cohort studies are rare, given the sample-size needed to identify an adequate number of cases with the identified pathogens of interest. This may explain why we only identified one cohort study for *S. dysenteriae* type 1 cases (HUS incidence was 8%,) and only two cohort studies involving STEC cases (median HUS incidence was 7.8%). We were able to identify eight case-control studies and concluded that 60.8% of HUS cases may be attributable to previous STEC infection.

We found fewer studies that examined the relationship between *S. dysenteriae* type 1 and HUS than STEC and HUS. While it is thought that *S. dysenteriae* type 1 tends to cause more severe HUS than does STEC and is associated with a higher mortality rate, we were unable to identify an adequate number of studies to quantify the relationship between *S. dysenteriae* type 1 and HUS. One possible explanation for the lack of case-control studies on *S. dysenteriae* type 1 is linked to the type of Shiga toxin (Stx) produced by this serotype. In humans, STEC that produces Stx 2 is more likely to be associated with the development of HUS than Stx 1 (22). While STEC may produce either Stx 1 or Stx 2, the Shiga toxin produced by *S. dysenteriae* type 1 is essentially identical to Stx 1 produced by *E. coli* (23). Another possible explanation for the lack of studies examining *S. dysenteriae* type 1-associated infections may be due to higher incidence rates in low- and middle-income countries compared to developed countries, resulting in fewer studies on *S. dysenteriae* type 1 and HUS (24,25). In addition, *S. dysenteriae* infection commonly occurs in outbreaks, especially among displaced persons after natural disasters and political crises, making prospective studies of incidence difficult (26). STEC is mostly an endemic infection agent, and incidence rates are less influenced by epidemics.

In this review, we were unable to control for heterogeneity among diagnostic tests in the included studies. It is possible that different positive values could have been obtained depending on whether serologic assays and/or stool tests were used for determining STEC infection. The incubation period for HUS ranges from three to eight days, with a median of 3-4 days after infection with STEC (27). The optimal window for the detection of STEC in stool samples is relatively narrow—about seven days after infection (28). Thus, serology tests as an adjunct to bacteriologic methods are important for detecting STEC antibodies and are considered the gold standard for reverse causation in HUS cases (9,29,30). Studies have shown that serological tests can provide evidence of infection for several weeks after the onset of diarrhoea (9).

In the studies that rely on stool cultures for the diagnosis of STEC, there could be false-negative stool cultures within HUS patients, thus reducing sensitivity. Using a combination of stool and serum tests to determine the presence of STEC generally gives a higher diagnostic yield (8,9,29). Of the included case-control studies, six used both faecal and serological tests while two used only stool cultures. In the case of multiple tests, we used the combined results or the more sensitive serology tests for our analyses. The two case-control studies (14,20) that did not include serology tests yielded lower rates of STEC infection (30-31.3%) than the studies that did employ serology tests (60-85.2%) (15-19,21). Both of these studies also discuss the lack of serologic testing as a limitation and note that they would expect higher rates of STEC identification with serology and polymerase chain reaction (PCR) results (14,20). Furthermore, we did not control for improving technologies for the detection of STEC or *S. dysenteriae* type 1 over the years in the included studies. This could confound the comparison of results of older studies with more recent studies. Initially, laboratory techniques for the detection of STEC were dependent on the presence of Vero cell cytotoxin or *E. coli* isolates in stool cultures which required a large number of isolates (31,32). Although cytotoxicity for Vero cells remains the gold standard, PCR is generally considered to be the most sensitive means of detection of STEC in faecal samples (33). Only two studies in this review reported the use of PCR for stool samples (12,13). Therefore, we might expect that all studies were biased toward more conservative rates of detection of STEC than would be observed if PCR had been utilized.

It is difficult to estimate case-fatality rates in low- and middle-income countries where the available data are weak, and many cases of HUS may go unrecognized. Clinical signs and symptoms may be vague; although HUS is often defined by renal impairment or failure, this symptom may not develop in all children (23). In underdeveloped areas, there is limited access to clinicians with the training and resources to diagnose the symptoms of HUS. The case-fatality rates for HUS are higher where there is no effective treatment. Care for patients that develop HUS is primarily supportive for anaemia, thrombocytopenia and its complications, and renal failure (23). Dialysis may be necessary for children with renal failure. Based on the existing studies of diarrhoea-associated HUS, authors estimated that after an average of four years, 3% developed permanent end-stage renal disease, and 25% suffered reduced renal function (34). The severity of the acute phase of HUS and the need for initial dialysis are strongly associated with a poorer long-term outcome (34).

### Conclusions

This review further quantifies the link between STEC or *S. dysenteriae* type 1 and HUS. Our results demonstrate that, while the incidence of HUS after STEC or *S. dysenteriae* type 1-associated infection is less than 10%, a significant proportion (60.8%) of HUS cases present with prodromal diarrhoea linked to these pathogens. If these bacterial infections progress to HUS, the risk of mortality increases. Since care-seeking is low and treatment options are fewer in developing countries, it is vital to reduce exposure to these pathogens to reduce diarrhoea-associated HUS cases. This can be accomplished by preventing faecal-oral transmission of STEC and *S. dysenteriae* type 1 through proper handwashing, improved sanitation, cooking meat thoroughly, and avoiding unpasteurized dairy products (35).

## ACKNOWLEDGEMENTS

The authors would like to acknowledge Peggy Gross, a public-health informationist with the Johns Hopkins Welch Medical Library, for her contributions to the literature search for this systematic review.
